# Transboundary determinants of avian zoonotic infectious diseases: challenges for strengthening research capacity and connecting surveillance networks

**DOI:** 10.3389/fmicb.2024.1341842

**Published:** 2024-02-16

**Authors:** Jeanne M. Fair, Nisreen Al-Hmoud, Mu’men Alrwashdeh, Andrew W. Bartlow, Sopio Balkhamishvili, Ivane Daraselia, Annie Elshoff, Lara Fakhouri, Zura Javakhishvili, Fares Khoury, Denys Muzyka, Levan Ninua, Jean Tsao, Lela Urushadze, Jennifer Owen

**Affiliations:** ^1^Genomics and Bioanalytics, Los Alamos National Laboratory, Los Alamos, NM, United States; ^2^Bio-Safety and Bio-Security Center, Royal Scientific Society, Amman, Jordan; ^3^Center of Wildlife Disease Ecology, Ilia State University, Tbilisi, Georgia; ^4^CRDF Global, Arlington, VA, United States; ^5^Department of Biology and Biotechnology, American University of Madaba, Madaba, Jordan; ^6^National Scientific Center, Institute of Experimental and Clinical Veterinary Medicine, Kharkiv, Ukraine; ^7^Department of Fisheries and Wildlife, Michigan State University, East Lansing, MI, United States; ^8^National Center for Disease Control and Public Health (NCDC) of Georgia, Tbilisi, Georgia

**Keywords:** wild birds, avian influenza, biosurveillance, pathogens, migration, science collaboration, zoonotic disease

## Abstract

As the climate changes, global systems have become increasingly unstable and unpredictable. This is particularly true for many disease systems, including subtypes of highly pathogenic avian influenzas (HPAIs) that are circulating the world. Ecological patterns once thought stable are changing, bringing new populations and organisms into contact with one another. Wild birds continue to be hosts and reservoirs for numerous zoonotic pathogens, and strains of HPAI and other pathogens have been introduced into new regions via migrating birds and transboundary trade of wild birds. With these expanding environmental changes, it is even more crucial that regions or counties that previously did not have surveillance programs develop the appropriate skills to sample wild birds and add to the understanding of pathogens in migratory and breeding birds through research. For example, little is known about wild bird infectious diseases and migration along the Mediterranean and Black Sea Flyway (MBSF), which connects Europe, Asia, and Africa. Focusing on avian influenza and the microbiome in migratory wild birds along the MBSF, this project seeks to understand the determinants of transboundary disease propagation and coinfection in regions that are connected by this flyway. Through the creation of a threat reduction network for avian diseases (Avian Zoonotic Disease Network, AZDN) in three countries along the MBSF (Georgia, Ukraine, and Jordan), this project is strengthening capacities for disease diagnostics; microbiomes; ecoimmunology; field biosafety; proper wildlife capture and handling; experimental design; statistical analysis; and vector sampling and biology. Here, we cover what is required to build a wild bird infectious disease research and surveillance program, which includes learning skills in proper bird capture and handling; biosafety and biosecurity; permits; next generation sequencing; leading-edge bioinformatics and statistical analyses; and vector and environmental sampling. Creating connected networks for avian influenzas and other pathogen surveillance will increase coordination and strengthen biosurveillance globally in wild birds.

## 1 Introduction

An estimated 1,855 (19%) of the world’s almost 10,000 bird species are migratory (Kirby et al., 2008). Migratory birds are defined as species that have substantial proportion of a regional or global population making cyclical movements beyond the breeding range, with predictable seasonal timing and is a physiologically demanding process (Klaassen et al., 2012). Migration distance can be from a few hundred kilometers, such as to lower elevations, to tens of thousands of kilometers. It may also link regions through zoonotic infectious diseases, as such diseases are known to be carried, transmitted, and propagated into new regions through migratory birds. Understanding which migratory species are important for the propagation of infectious diseases is the crucial first step in optimizing biosurveillance for especially dangerous pathogens. With the current situation with highly pathogenic influenzas and the dramatic increase of spillovers back and forth between wild birds and mammals, it has become more important than ever to strengthen surveillance in wild birds globally. The objective of this review is to address what it takes to strengthen wild bird biosurveillance capabilities into new regions and increase coordination around the world.

## 2 Strategy for global influenza surveillance

Recently, there have been numerous calls to increase biosurveillance and monitoring of wild birds for highly pathogenic avian influenza (HPAI) and other pathogens (Adlhoch et al., 2022; Hill et al., 2022; Günther et al., 2023). In addition, there is already ample evidence that continuous surveillance of wild birds can increase detection of pathogens and help answer questions for a better understanding of the ecology of avian zoonotic infectious diseases (DeLiberto et al., 2009; VanDalen et al., 2010; Gamarra-Toledo et al., 2023). To identify ways to optimize wild bird surveillance for influenza, Machalaba et al. (2015) reviewed responses to a World Organization for Animal Health (OIE)–administered survey, as well as other sources. They found that at least 119 countries conducted avian influenza virus surveillance in wild birds during 2008–2013, which was mostly focused on limited subsets of influenza viruses. This team also found that 23.9% of 46 OIE-member responding countries reported active (live birds) and passive (dead birds) surveillance activities, 30.4% reported active surveillance only, and the same percentage conducted passive surveillance only (Machalaba et al., 2015). The remaining 15.2% reported conducting no surveillance activity of wild birds. One of the primary findings of this review was that surveillance activities used different sampling methodologies, and there was a lack in coordination and reporting of metadata for samples. Similarly, a review by Hoye et al. (2010) of avian influenza surveillance conducted during 1961–2007 suggested that unstandardized sampling remains a continuous challenge for global avian influenza virus surveillance, as well as other zoonotic pathogens in wildlife (Hoye et al., 2010). Lastly, Duan et al. (2023) calls for the establishment and enhancement of interdisciplinary and cross-sectoral coordination and cooperation among medical, veterinary, and public health institutions, and the sharing of surveillance information for timely alerts.

Machalaba et al. (2015) point out that surveillance efforts should move past just searching for HPAI to looking for the full viral diversity of influenzas virulence and subtypes. This would add information to international reporting requirements that can provide additional understanding of transmissibility, pathogenicity, and host range, among other factors. Additionally, coordinated efforts for sharing data and information on avian health and condition in migratory birds is essential for understanding and predicting avian influenza and other pathogens in birds.

### 2.1 Active surveillance vs. reporting

Currently, while many countries around the world have national wildlife health (NWH) surveillance programs, many do not, or the programs vary in scope and size. There are many different types of surveillance investigations that are possible depending on the overarching goals for national or regional coordinated effort. Lawson et al. (2021) highlight 10 challenges for creating a NWH surveillance program in a country with key recommendations for each. Here, we offer recommendations that are designed for all levels of pathogen surveillance activities in migratory wild birds, with a focus on researching the ecology of zoonotic infectious diseases, as well as traditional monitoring in wild birds.

Active infectious pathogen or disease surveillance in wild birds is important for several reasons, including detecting and responding to emerging diseases early, tracking the spread of diseases, identifying potential risk factors, assessing the impact of diseases on wildlife populations, and lastly, implementing control measures. Active infectious disease surveillance in wildlife can be done through a variety of methods (Lawson et al., 2021). Wildlife disease sampling is the process of collecting samples from wildlife, such as blood, tissue, and feces, to test for the presence of pathogens. This information can be used to identify the prevalence of pathogen in otherwise apparently healthy wildlife populations, and to track specific pathogens or look for newly emerging microbes. Pathogen surveillance is the process of collecting data over time to track the occurrence of diseases in wildlife populations through past exposure using serology or detecting active infections through various methods in samples from individuals. This information can be used to identify trends in disease occurrence and to assess the effectiveness of control measures and the dynamics of the disease system (Plowright et al., 2019). Disease reporting or syndromic surveillance, however, is the process of collecting information about diseases that have been observed in bird populations using outbreak events that lead to mortalities in wildlife (Lawson et al., 2021). While this information can be collected from sources such as hunters and wildlife managers, it is most often collected in response to a mortality event, such as avian influenza in waterfowl or West Nile virus deaths in corvids.

**Figure 1:**
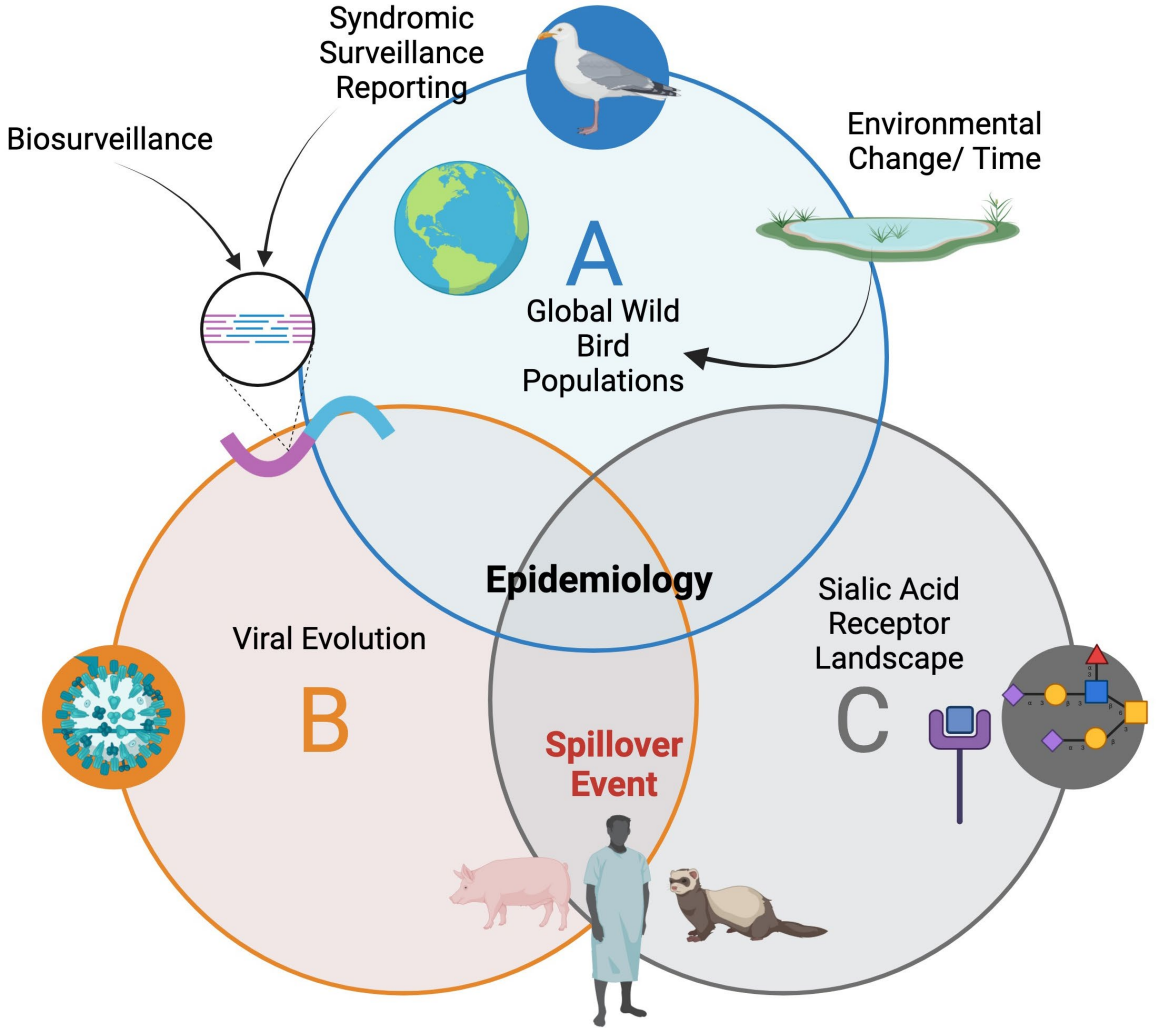
The epidemiology landscape for avian influenzas in wild birds and the need for connection with the multidisciplinary teams.

Both wildlife disease sampling in healthy appearing populations and syndromic surveillance are important for tracking viral evolution as in the case of avian influenzas (Figure 1). Understanding both viral evolution and the sialic acid receptor landscape in reservoirs and other potential hosts is crucial for forecasting spillover events. Counties around the world and regions need both active surveillance in addition to reporting of sick or dead birds to better understand infectious diseases in migratory birds and to track the emergence and evolution of zoonotic pathogens.

### 2.2 Importance of migratory birds in pathogen global distribution

Migratory birds play a crucial role in the transmission of infectious diseases, serving as both hosts and vectors for various pathogens. The intercontinental movement of wild birds across vast geographical areas can provide opportunities for the exchange of pathogens between different regions and populations (e.g., Dusek et al., 2009; Araujo et al., 2018; Ayadi et al., 2019). The best known, and most documented example, is the H5N1 avian influenza virus that has been detected in wild birds during migration, contributing to the global dissemination of the virus (Olsen et al., 2006). As in the new H5N1 clade, migratory birds introduce novel strains of pathogens to new areas, leading to potential outbreaks among local wildlife and domesticated animals. Understanding the dynamics of infectious disease transmission involving migratory birds is essential for implementing effective surveillance and control measures to mitigate the risks of zoonotic spillover events (Gaidet et al., 2012; Verhagen et al., 2021). It is important to note that the movement of all avian influenzas globally is through wild birds.

The other most documented examples of a zoonotic pathogen spread geographically by wild birds is West Nile virus (WNV). The expansion of the range for WNV in the United States from 1999 to 2000 was along the Atlantic seaboard, a common migration route for many bird species that have summer ranges in the northeastern United States. From 2000 to 2003, WNV moved west across North America faster than predicted for contiguous spread of infection by a mosquito (Reed et al., 2003). Recently, there is new concern for migratory birds spreading multi-drug resistant genes in bacteria, increasing this risk of these newly emerged zoonotic bacteria (Shah et al., 2022). While migratory birds have often been seen as a main cause for contaminating water sources with the zoonotic parasites, Cryptosporidium and Giardia, new evidence points out that this is probably overestimated (Egan et al., 2023).

## 3 The history and current situation of avian influenzas

Since 2003 through December 1, 2023, 880 humans have been infected by H5N1 in 20 countries and maintained a 56% mortality rate as reported to the Whole Health Organization’s Global Influenza Program (WHO, 2023). In 2005, H5N1 was found to have killed a population of over 6,000 brown-headed geese (*Anser indicus*) at Qinghai Lake in China, which was the start of this strain entering the environment (Wang et al., 2008). Over the next 3 years, the H5N1 strain moved across Asia and into Europe, and for the next 18 years there were no significant outbreaks. Then in May 2021, the H5N1 influenza virus was detected in wild red fox cubs (*Vulpes vulpes*) at a rehabilitation center in the Netherlands during an outbreak of HPAI of H5 clade 2.3.4.4b in wild birds (Rijks et al., 2021). This was one of the first indications reported that HPAI H5 clade 2.3.4.4b viruses may spillover from birds to mammals. With the number of such spillover events increasing, spillovers into mammals from avian influenza can now be considered commonplace and not rare and sporadic. It is due to this increase in spillovers and reverse spillovers, that surveillance of wild birds is more important than ever.

Highly pathogenic avian influenza viruses of the H5N1 subtype [clade 2.3.4.4b of the goose/Guangdong (Gs/GD) lineage] have quickly spread across North America since detection in December 2021 (Wille and Barr, 2022). Not only has this H5N1 clade 2.3.4.4b caused many mortality events in several species of wild birds and domestic poultry, but it has also been found to be transmissible to mammals, renewing concern for it becoming a pathogen of pandemic potential (Gilbertson and Subbarao, 2023). Reports of H5N1 transmission to mammals include harbor seals (*Phoca vitulina*) and gray seals (*Halichoerus grypus*) in New England (Puryear et al., 2023), and several mesocarnivore species such as red foxes, striped skunks (*Mephitis mephitis*), and mink (*Neovison vison*) in Canada (Alkie et al., 2023).

In 2020, it is thought that a subclade of the 2.3.4.4b influenza virus (that originally evolved in 2014–2015 H5Ny) reassorted or paired with an N1 neuraminidase (Caliendo et al., 2022). This new virus then spread to many parts of the world, including Africa, Asia, Europe (Xie et al., 2023), and North and South America (Kandeil et al., 2023). The new 2.3.4.4b clade has now devastated wild bird populations and caused outbreaks in domestic poultry around the world (Leguia et al., 2023). Notably, this clade has also caused infections in various small and large mammals, including terrestrial to marine mammals with different ecologies (Gilbertson and Subbarao, 2023). Most of these have been “dead end” infections and are attributed to direct contact due to scavenging infected birds. However, an outbreak at an American mink farm in Spain (Agüero et al., 2023) marks the first H5N1 infections potentially involving mammal-to-mammal transmission. Not only has this increased concern for the zoonotic potential of H5N1 avian influenza, but it has also increased the call for more biosurveillance and monitoring of wild birds (Gilbertson and Subbarao, 2023).

In addition to the outbreaks in wild birds and mammals, the current H5N1 situation has devastated poultry populations in North America and Europe. The United States Department of Agriculture confirmed highly pathogenic avian influenza (HPAI) in a commercial flock in the United States on February 8, 2022. Since the outbreaks began in early 2022, poultry outbreaks across 47 US states have impacted a record 75.4 million birds through December 2023 (USDA, 2023). In February 2020, the Saudi Arabian government reported an outbreak of the highly pathogenic H5N8 virus on a poultry farm. Since that time, H5N8 has been identified in numerous countries and continents, leading to the subsequent culling of millions of birds (Reuters, 2023).

## 4 Wild bird taxon groups, ecology, and associated pathogens (host range), differences in surveillance efforts

### 4.1 Waterfowl

Migratory waterfowl in the order Anseriformes play a crucial role in the epidemiology of avian influenza. These birds, including ducks, geese, and swans, are the natural reservoirs for many influenza A viruses (Blagodatski et al., 2021). The high genetic diversity of avian influenza viruses in waterfowl populations contributes to the adaptability and potential for reassortment, as well the important role of the environment (Stallknecht and Brown, 2008). It is well established that influenza prevalence peaks in waterfowl in the late summer and early fall, particularly in some dabbling ducks (Kent et al., 2022). Waterfowl are the most sampled and studied birds for avian influenzas and most surveillance programs are focused on waterfowl. There are numerous good reviews for the role of waterfowl in avian influenza propagation (Henaux and Samuel, 2011; El Zowalaty et al., 2013; Hill et al., 2022).

Moreover, waterfowl are implicated in the transmission of other viral pathogens, such as avian paramyxoviruses (e.g., Hinshaw et al., 1985; Klink et al., 2023) and various avian coronaviruses (e.g., Chu et al., 2011; Sharshov et al., 2023). Waterfowl are also important for the transmission bacterial pathogens including Salmonella, Campylobacter, *Chlamydophila psittaci*, Clostridioides difficile (formerly *Clostridium difficile*), and Pseudomonas (Benskin et al., 2009). This is particularly true for waterborne bacteria and contamination of waterways (Chung et al., 2018). The movements of migratory waterfowl across continents can facilitate the geographic spread of these pathogens and are often referred to as synanthropic species that are ecologically associated with humans and agricultural (Shriner and Root, 2020). The intricate interplay between the waterfowl, pathogens, and the environment underscores the importance of a holistic approach to avian health that considers the ecology and behavior of these birds.

### 4.2 Shorebirds and gulls

Shorebirds are in the order Charadriiformes, which contains three suborders: (1) Scolopaci (sandpipers, snipes, phalaropes, and jacanas), (2) Charadrii (plovers, oystercatchers, and stilts), and (3) Lari (gulls, terns, auks, and skuas). The prevalence of highly pathogenic avian influenza virus infection in shorebird species sampled globally is typically considered low (approximately 1%) compared to the prevalence in ducks (approximately 10% globally with migration season peaks of 20–60%; Olsen et al., 2006; Munster et al., 2007). The exception to this finding is the ruddy turnstone (*Arenaria interpres*), which has a consistently high AIV prevalence (10%) during spring migration at Delaware Bay, United States (Kawaoka et al., 1988; Hanson et al., 2008; Stallknecht et al., 2012).

Gulls have been found to be important in the movement and propagation of both low (LPAI) and HPAI viruses. In addition to all H and N subtypes being detected in gulls, H13 and H16 subtypes are considered “gull-adapted” (Arnal et al., 2015). Experimental infection and laboratory studies have showed high morbidity and mortality following infection with HPAIV H5N1 subtype in herring gulls (*Larus argentatus*) (Brown et al., 2008), black-headed gulls (*Chroicocephalus ridibundus*) (Ramis et al., 2014), and laughing gulls (*Larus atricilla*) (Perkins and Swayne, 2002). In recent outbreaks of HPAI since 2021, gulls have always been found among both live and dead, positive-testing wild birds during consecutive outbreaks across Europe (Adlhoch et al., 2022, 2023).

### 4.3 Passerine landbirds

Passerine landbirds in the order Passeriformes, commonly known as songbirds, have gained attention in recent years as potential reservoirs and vectors for zoonotic pathogens—particularly West Nile virus (WNV). Passerines, with their extensive migratory patterns and use of essential every ecosystem, can serve as important indicators of environmental health (Morrison, 1986). In North America, Rosenberg et al. (2019) report wide-spread population declines of birds over the last 50 years resulting in the cumulative loss of billions of breeding individuals across a wide range of species and habitats. The decline is particularly precipice in the landbirds that have declined approximately 27% (Rosenberg et al., 2019). In 2022, BirdLife International’s long-running State of the World’s Birds also highlights the global trend for the rapid and widespread bird population decline (Birdlife International, 2022).

Passerine birds are susceptible to a range of infectious agents, including viruses, bacteria, fungi, and parasites that can also have profound effects on passerine populations. One disease example is avian pox, caused by avipoxviruses, which affects passerines worldwide. Avian pox manifests as wart-like growths on the skin, beak, and feet, leading to impaired vision, feeding difficulties, and, in severe cases, death. A recent review highlights the prevalence of avian pox passerine and non-passerine populations, emphasizing the need for continued monitoring and research to understand the dynamics of the disease in these birds (Williams et al., 2021).

Furthermore, migratory passerine birds can serve as important reservoirs and vectors for various infectious diseases with implications for both avian and human health (Hubálek, 2004). Passerine birds are considered the primary reservoir for WNV which is transmitted by mosquitoes and affecting both birds and mammals. Numerus studies in North America, underscore the role of passerines as amplifying hosts for WNV, contributing to its spread in North America (Kilpatrick et al., 2006; Levine et al., 2017). West Nile Virus and its mosquito vectors, are intricately linked to climate change, increasing the complexity of the disease (Paull et al., 2017; Gorris et al., 2023; Heidecke et al., 2023).

Understanding the epidemiology of infectious diseases in passerine birds is crucial for conservation efforts and public health, as these birds can influence the transmission dynamics of pathogens in both avian and human populations. West Nile virus has had significant impacts on bird passerine populations (LaDeau et al., 2007), but that may not be long-lasting (Kilpatrick and Wheeler, 2019). Kilpatrick and Wheeler (2019) found evidence that many wild bird species and populations have recovered from the initial WNV impact, but a few passerine species have not. Why a few species, such as the purple finch (*Haemorhous purpureus*) in California, have not recovered remains a mystery, requiring additional research. Passerine birds, particularly corvids such as crows and magpies, are known to be highly susceptible to WNV infection and often serve as amplifying hosts, contributing to the virus’s transmission cycle (Wheeler et al., 2021). Understanding the dynamics of WNV in passerine birds is essential for monitoring and managing the spread of the virus, as well as for implementing effective public health measures to mitigate the risk of human infection.

## 5 Ticks and wild birds and associated pathogens

As with mammals and lizards, birds can be parasitized by ticks and infected with tick-borne pathogens, which include bacteria, viruses, and parasites. Birds are known to play important roles in the maintenance of enzootic cycles of zoonotic tick-borne pathogens, such as those that cause Lyme borreliosis (Hanincová et al., 2003; Comstedt et al., 2006; Hildebrandt et al., 2010). Although a relatively small proportion of migrating birds may be parasitized by ticks and/or tick-borne pathogens at any time, the large number of birds migrating en masse can result in a non-negligible number of introduced ticks, especially when birds may congregate at stopover sites. Migrating birds can disperse ticks and tick-borne pathogens over large distances and beyond geographic features (e.g., rivers, deserts, mountains, and seas) that present dispersal challenges to terrestrial wildlife. For example, Neotropical and African ticks (including ones infected with pathogens), have been recorded on northward migrating birds in the United States (Mukherjee et al., 2014; Cohen et al., 2015) and Europe (Hasle, 2013; Hoffman et al., 2023), respectively.

Multitudes of exotic ticks introduced over millennia have failed, however, because of a mismatch of suitable abiotic conditions and/or preferred wildlife host species. For example, in early studies conducted nearly 70 years ago, Hoogstraal and Kaiser (1961) characterized the tick fauna infesting birds migrating through northern Egypt during both fall and spring (Hoogstraal and Kaiser, 1961; Hoogstraal et al., 1963). It was revealed that northward migrating birds could be infested with and therefore could disperse *Hyalomma rufipes* ticks, which are native to central and southern Africa. Because these ticks will feed on the same host individual for both larval and nymphal life stages, they will stay with the host for approximately 26 days, allowing these ticks to be transported far from their endemic range. For *H. marginatum*, a related tick species that is endemic to the Mediterranean Basin, this is also true. Thus, spring migrating birds can disperse engorged nymphs of both species, which will molt successfully into adults only if the subsequent months are warm enough for long enough, given development is a temperature-dependent process (Estrada-Peña et al., 2021; Estrada-Peña, 2023). In recent years, however, adult *H. marginatum* and *H. rufipes* ticks have been detected more frequently in northern latitudes, and this may be due to the warming climates (Chitimia-Dobler et al., 2019; Grandi et al., 2020, 2023; Uiterwijk et al., 2021). This is of public health concern because both ticks are known vectors of Crimean Congo Hemorrhagic Fever virus (CCHFv), which can cause severe disease with a 30% fatality rate (Hoogstraal et al., 1979). In addition to CCHFv, *Hyalomma* spp. ticks can carry pathogenic rickettsia and *H. rufipes* ticks also can carry Alkhurma Hemorrhagic Fever Virus (Hoffman et al., 2018). Although Hoogstraal et al. (1963) studied the tick infestations of southward migration of birds through northern Egypt, there are far fewer studies recording the southward migration of ticks from Europe into Africa. Southward migrating birds are infested with a higher proportion of *Ixodes* spp., ticks, including *I. ricinus* (the vector for Lyme borreliosis bacteria). There are populations of *I. ricinus* in North African countries, which appears to be the southern limit of this temperate tick species.

To infer whether the host may have infected a feeding tick or whether the feeding tick was already infected (and may be infecting the host), one should note the life stage of the feeding tick and whether the microbe is one that could be vertically transmitted between tick generations. If the microbe cannot be vertically transmitted from adult females to larval offspring, then detection of the microbe in a feeding larva indicates that the larva acquired the microbe from the host, and therefore the host is systemically infected. It is also possible that the host is not systemically infected, but rather that the larva acquired the pathogen from an infected tick (another larva or nymph) feeding nearby in time and location. This latter process is called co-feeding and/or non-systemic infection and occurs with tick-borne encephalitis virus and *Ixodes ricinus* ticks (e.g., Labuda et al., 1993; Tsao, 2009). Detecting an infected nymphal tick may not permit deduction of the source of infection, but regardless, it suggests that the host likely has been exposed to the microbe, and that it is transporting and dispersing infected ticks. Without more knowledge about transmission cycles, one might not know how best to interpret pathogen data and may be confused by contradictory data provided by tick and host tissue samples obtained at the same capture event (Korobitsyn et al., 2021).

Furthermore, the more knowledge that exists about the current geographic range of tick species as well as the activity periods of each life stage, the better one can infer whether a tick that is attached to a bird is one that is locally endemic and may have been locally acquired or whether it is an exotic tick that is being introduced. In this regard measuring the engorgement status of the tick (e.g., the scutal index) that has been sampled from a non-resident bird may help indicate for how long the tick may have been feeding (e.g., 1 v. 4 days) and therefore perhaps the origin of the encounter between the tick and the bird host (Couret et al., 2017). Finally, as ticks often can acquire microbes from the host even if they are not competent reservoirs, assaying an attached tick for the presence of a microbe may help infer whether the host is infected and therefore introduce the microbe to subsequently feeding local ticks (Hamer et al., 2010). Training researchers to collect and identify ticks on migratory birds can be added to any biosurveillance activity, or collaborations with tick experts and other laboratories can be established.

## 6 What does it take to do surveillance of wild birds?

**Figure 2:**
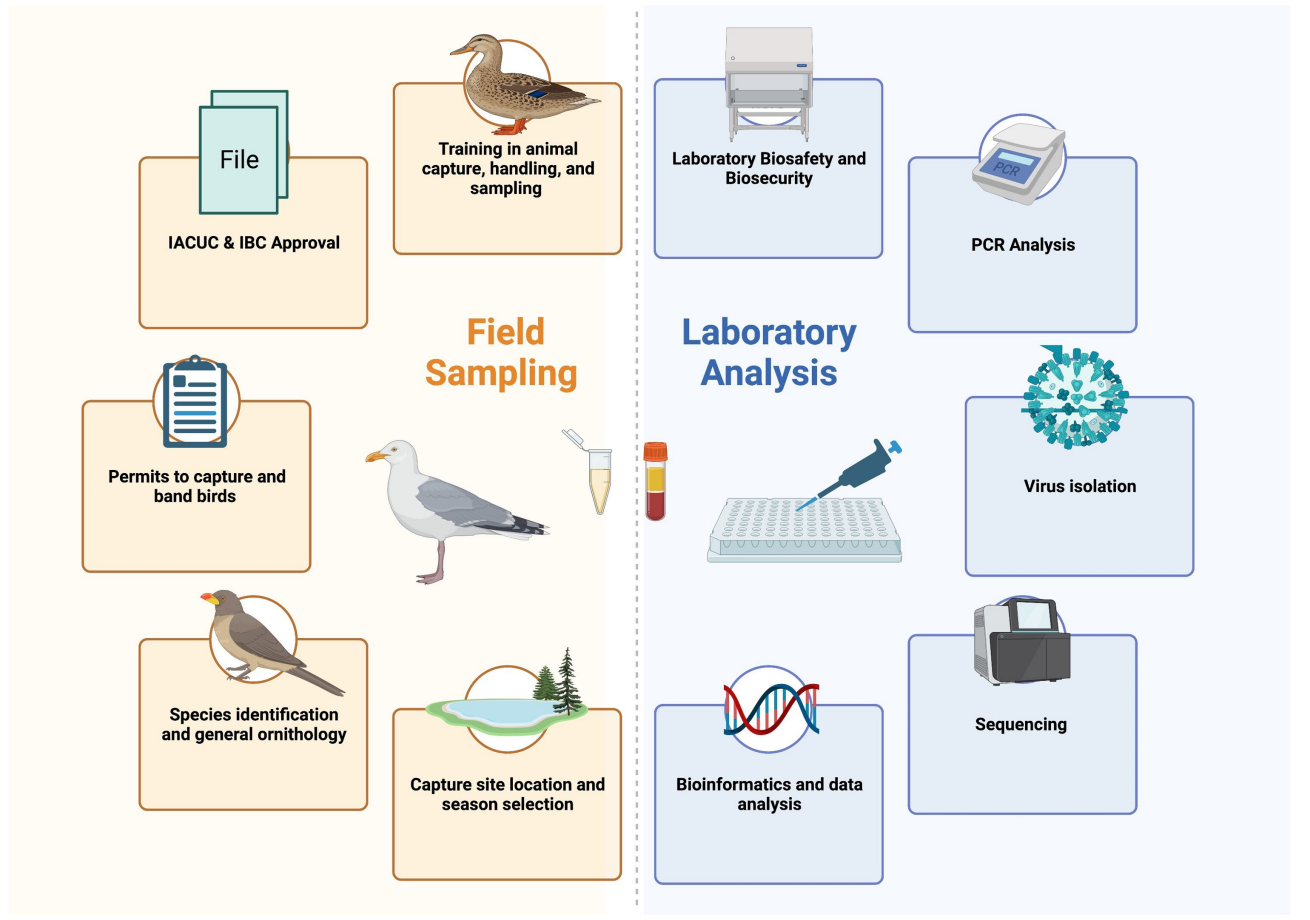
Each of the different aspects that field sampling and laboratory analysis entail. Field sampling and laboratory analysis can be done completely separately, but there is better integration and data quality when each component work together to troubleshoot and ask better questions together.

Understanding the complex intertwined systems of pathogen transmission for zoonotic avian diseases is challenging, time consuming, and costly. While direct biosurveillance is important to detect and diagnose outbreaks to rapidly respond to and reduce the threat, research to understand the host-reservoir-pathogen-environment system is also crucial for curtailing future epidemics. Coordination across many fields and government sectors is required. Planning research to understand the role of migratory birds in zoonotic diseases is difficult to coordinate between field and laboratory experts, and likewise, ensuring that all training for animal capture and biosafety is developed, as well as having the appropriate permits, approvals, and government requirements are in place also present logistical challenges (Figure 2). Protocols are required for both field and laboratory work, with emphasis on biosafety in both environments. The overwhelming challenge is in training the skills required to go from sample to sequence and relies on mentorship to strengthen these new capabilities in the field and the laboratory.

### 6.1 Permits, approvals, and international agreements

In nearly every country in the world, a permit is required to capture, handle, and sample wild birds. Although wildlife laws vary in each country, permission must be granted by the national government, usually the ministry of agriculture or the environment, and sometimes from a state-or regional-level agency as well. Prior to beginning sampling, all permits related to the protection of natural bird populations and the environment and to providing oversight of compliance for trapping and handling birds for research *must be in place*. Often, even if no birds are captured and sampled and only environmental samples are taken, a research permit is required. A single project can involve multiple permits at the national and state levels, and it can take months to obtain any one permit, which may well be overwhelming (Paul and Sikes, 2013). However, permits, such as those resulting from the Migratory Bird Treaty Act (MBTA) of 1918, are intended to ensure population stability of all protected migratory bird species. The MBTA implements four international conservation treaties that the United States made with Canada in 1916, Mexico in 1936, Japan in 1972, and Russia in 1976. For example, in the United States, federal scientific collecting permits are required for birds, parts of birds, eggs, and/or nests for any species covered by the Migratory Bird Treaty Act. This includes blood and tissue samples, feathers, stomach and crop contents, and cloacal and tracheal swabs. In addition, if threatened or endangered wild bird species are to be sampled, the Washington Convention on International Trade in Endangered Species of Wild Fauna and Flora or “CITES” will apply for the capturing and handling of these species. Lastly, if sampled blood is left over and found to be negative of pathogens, blood can be offered or shared via the Nagoya Protocol on Access to Genetic Resources and the Fair and Equitable Sharing.

Agreements to use government-owned lands to capture birds may also be required. In many countries, remaining natural areas where birds congregate are owned by the national government or regional agencies. Permission to conduct research will be required for these areas and may involve coordinating with government or agency personnel for access or for help with sampling. In addition to getting all stakeholders onboard, a documented understanding ensures that all officials are aware of the research work and support the researchers.

International agreements will most likely be required, and especially in the exchange of monies between governments for research. There may be many public policy requirements that are applicable to applications from foreign organizations. For example, in May 2023, the United States National Institutes of Health (NIH) released updated grants policy guidance requiring that each primary awardee institution impose on foreign entity subawardees an “obligation to provide to the prime awardee all relevant research records (including data and lab notebooks), and to do so at an agreed-upon frequency of not less than every 3 months” (Barnes et al., 2023). Other requirements for funding between foreign entities may be required regardless of the countries in partnership, and it is important to understand the requirements early in the process.

### 6.2 Institutional animal care and use committee

An Institutional Animal Care and Use Committee (IACUC) is responsible for ensuring the ethical and humane care and use of animals in research in the United States. IACUC approval is required for all research involving animals, including wildlife, and is often required for publishing animal study results in scientific journals. Not every country or institution has an IACUC, and projects involving wildlife regularly face issues where guidance requirements may not be available (Leland et al., 2019).

Still, there are several reasons why IACUC approval is important for wildlife research. IACUCs have expertise in animal welfare and can help to ensure that animals are treated humanely, that research is conducted in a way that minimizes pain and suffering to animals, and that research is conducted in a way that is scientifically sound. While government ministries or organizations leading biosurveillance efforts for birds may not require IACUC approval, sampling for other research questions will require animal care approval. While most IACUC resources are geared toward laboratory animals, there is specific guidance for working with wild birds, such as the Guidelines for the Use of Wild Birds in Research (Fair et al., 2023) that can be used by both researchers and IACUCs. Capacity building may entail setting up an IACUC in a region or country, and the approval process will help ensure adequate sampling methods and samples sizes. If sampling is only considered for specific pathogens monitoring, then IACUC approval may not be required. IACUC members are required to complete annual trainings specific to IACUC and most IACUCs then require additional training for researchers to understand proper animal care and the role of IACUCs.

### 6.3 Geospatial and location differences

Differences in location and habitat not only affect pathogen transmission but are also important for tracking from a sampling and monitoring perspective. These are the same differences that can affect the transmission of waterborne diseases. Habitat differences for passerine birds also vary, but not as much as waterfowl or shorebirds to influence mist net monitoring methods. Historic data on the environmental conditions, habitat, and climate patterns will provide insight into how locations have changed over time. Collecting new metadata at each sampling location is important for detecting differences in wild bird use and impacts on pathogen prevalence.

### 6.4 Avian host health

Estimating and measuring the health and condition of wild birds has been a staple of ornithology for over 70 years. Estimating the health of an individual bird has been used to measure the impacts of stress, environmental contamination, migration, reproduction, infectious diseases, and numerous other variables. New methods and techniques have become available to measure a physiological parameter related to metabolism, nutritional status, hormones, and immune function in wild birds (Xiong et al., 2023). Understanding the current physiological health status can be crucial for insight into how bird may be susceptible to infections, co-infections, and the role of superspreaders in potential outbreaks (Jankowski et al., 2013; Fritzsche McKay and Hoye, 2016). In addition, studying physiological variation in response to biotic and abiotic stressors can help us to understand and predict how animals can cope with exposure to viral and bacterial pathogens under stressful conditions including potential effects of global change (Fuller et al., 2012). While most biosurveillance initiatives do not measure or report on the condition indices for wild birds, overall health is an important variable to consider when asking more in-depth questions about the ecology of a wild bird disease system.

### 6.5 Age and sex impacts on infection probabilities

To understand the epidemiology of a pathogen in a wild bird population, information on the ages of sampled animals is important, as the age of seroconversion or the age of infection can be used to estimate transmission rates and the location of infection. Seroconversion in juvenile birds can show that a pathogen is likely to be persistent and endemic in that population’s breeding grounds. This is particularly important information for avian influenza in northern arctic regions, which are known to harbor influenzas at high levels in waterfowl and shorebirds (Wilson et al., 2013; Meixell et al., 2016). Clearing infections in birds prior to migration historically kept influenzas or other pathogens from spreading (Hoye et al., 2011). Being able to correctly age captured birds requires specific training and can be complicated (Norevik et al., 2020; Pyle, 2023), but can give important information on infection dynamics in juveniles in a species.

Understanding the epidemiology of a pathogen in a wild bird population also requires knowledge of how the sexes of the species may differ in exposure to, infection from, and recovery from the pathogen. Foraging habits and locations may differ by sex, and thus exposure to a pathogen, stress-induced immunocompetence, or the general ability to clear infections may also differ. In humans, the H7N9 avian influenza greatly impacted men over women, first, it was thought that this was because men are most often poultry workers who have more exposure to birds (Rivers et al., 2013). However, it was later confirmed that H7N9 avian influenza virus infection in men is associated with testosterone depletion (Bai et al., 2022). It has been shown that high-dose H1N1 infection reduces testosterone levels in male (but not female) mice (Tuku et al., 2020). Similarly, aged male mice with low testosterone levels were reported to undergo elevated pulmonary inflammation and severe disease upon H1N1 influenza virus infection compared to young male mice (Vom Steeg et al., 2016). The mechanisms for this are not entirely clear and currently, there are no published studies investigating avian influenza infections in wild birds with regard to testosterone. While this information can be obtained from mortality events in wild birds, the data are rarely reported. To obtain a better understanding of epidemiology in wild bird populations, both surveillance and reporting in wild bird monitoring should include and record both the sex and age of infected or sampled birds. Many bird species are not sexually dichromatic and not be aged phenotypically, but can be sexed using DNA from a drop of blood on a FTA® card for example Gutiérrez-Corchero et al. (2002).

### 6.6 Sample collection techniques

While there are entire books, guidelines, and journal issues written on methods for sample collection in wild birds for infectious diseases (Martin et al., 2006; Mazzamuto et al., 2022), there remains no standardization for samples collected from wild birds or the environment near wild bird populations. The most common samples collected in live wild birds are cloacal and tracheal or oropharyngeal swabs, and blood for serology, immunology, and overall health. Environmental sampling includes samples or swabs taken from feces, mud, water near or around birds, and feathers (Hood et al., 2021). For areas near domestic birds, surfaces likely to be contaminated with viruses, such as cages in markets or processing surfaces, may also be swabbed. For over 4 decades, avian influenza surveillance programs have used and cultured water and fecal samples from wild bird and domestic duck habitats to detect influenza viruses (Hinshaw et al., 1979).

Early studies in waterfowl showed that cloacal swabs yielded a larger number of isolates of avian influenza viruses than did respiratory tract swabs (Krauss et al., 2013). Traditionally, avian influenza surveillance has focused on collecting cloacal or environmental fecal samples. However, HPAI H5N1 virus has been found to replicate with higher titers in the respiratory rather than the gastrointestinal tract in waterfowl (Sturm-Ramirez et al., 2005; Brown et al., 2006), prompting the collection of respiratory samples in addition to cloacal swabs from wild birds. Krauss et al. (2013) completed a large biosurveillance study of 1,036 wild ducks in Alberta, Canada to compare respiratory tract and cloacal swabs for avian influenzas. The authors were able to determine that of the 28 HA–NA subtype combinations detected in the wild ducks, three were found only in the respiratory tract (H3N5, H3N6, and H4N5), nine were found in both the respiratory tract and the cloaca (H1N4, H3N8, H3N9, H4N3, H4N4, H4N6, H4N8, H7N3, and H7N8), and the remaining 16 combinations in cloacal samples only (Krauss et al., 2013). Additional information from comparing respiratory and cloacal swabs could help our understanding of the role of respiratory shedding in the spread of avian influenzas. Therefore, for an overall biosurveillance program for avian influenza, it is recommended to take both a cloacal and a respiratory swab for all sampled individuals.

Training to collect different types of samples from a bird first requires training in proper handling and sampling for each of the three primary bird taxon groups. This is particularly true for taking blood samples in wild birds. Smaller birds may require more delicate, yet firm handling with sampling, and some types of sampling may not be appropriate for smaller bird species under 20 g. In wild birds, blood is mostly obtained from the brachial and jugular veins, although blood can also be collected from the metatarsal or leg veins, toenails, or the heart (Owen, 2011). It is important to consider that current guidelines suggest no more than 1% of a bird’s body mass should be collected per blood collection event (McGuill and Rowan, 1989). For a complete review of blood sampling techniques and blood smear methods, see Owen (2011).

### 6.7 Biosafety in the field

Due to the propagation and increase in zoonotic infectious diseases in wild birds such as HPAI and West Nile virus, considerations for protecting the health of researchers are highly important. While non-disease sampling bird capture and ringing programs do not require extra personal protective equipment (PPE), the identification of bloodborne pathogens in birds means nitrile or similar gloves are recommending when taking blood samples. For avian influenza sampling, different government agencies have different regulations for sampling waterfowl and shorebirds, which include double nitrile gloves and precautions when sampling. Recommendations for PPE for field personnel who handle apparently healthy wild birds in areas where HPAI is not suspected are different than recommendations for personnel handling sick or dead birds associated with a morbidity/mortality event or where HPAI is known to be recently located (Martin et al., 2006). In addition to protecting human health, it is important to protect birds from each other and not spread any infectious disease to a new location. Training for the appropriate field biosafety for the different situations is crucial establishing a surveillance program.

## 7 Laboratory analysis

Samples for laboratory assays must be collected, selected, preserved, transported, and stored in proper ways and with highly important considerations. The first consideration is to use sterile sampling tools (swabs, tubes, reagents, needles, etc.) to prevent DNA/RNA contamination between samples and infection of the animal. Second, a disinfectant (such as alcohol or bleach) must be applied to tools and processing areas before, during, and after the sampling process. Biological samples for laboratory analysis must be preserved in proper conditions to ensure quality and integrity. All specimens for virus detection assays must be stored in cryovials containing reagents, such as RNA*later* or newer, cheaper agents, to maintain RNA integrity (Wille et al., 2018). Similar protocols for sample storage and transport should be in place for DNA for bacteria identification and is decision for sampling protocols. For ensuring cold chain, a liquid nitrogen-containing tank should be used in the field to store the samples until they are transported to an appropriate laboratory freezer. Operating a diagnostic laboratory should include all biosafety precaution and certifications, such as annual biosafety cabinet testing, and trainings for personnel in safe operation of all instruments.

Timely and accurate detection of avian influenza viruses and other zoonotic pathogens is crucial for implementing effective control measures and preventing additional viral evolution. PCR and serology are the two key diagnostic tools employed in pathogen surveillance. PCR enables the amplification of specific viral nucleic acids, providing the most sensitive and rapid detection of the virus in clinical samples. To detect and quantify RNA, quantitative reverse transcription PCR (RT-qPCR), is used. On the other hand, serological assays, such as enzyme-linked immunosorbent assay (ELISA), detect antibodies produced in response to pathogen infection (e.g., avian influenzas; Spackman and Killian, 2020) offering insight into the history of exposure within individuals. Serology can be more difficult for wild birds if the ELISA reagent kits were developed for poultry and may not be reactive with taxonomically different.

While PCR directly identifies the presence of the virus, serology provides information about the immune response, aiding in understanding the dynamics of virus circulation. Both techniques complement each other and provide different information for current outbreak, as well as the range of a pathogen for risks maps. In some regions of the world, the newest or most sensitive PCR and ELISA kits may not be available, or they may be difficult to obtain. In influenzas, the relatively conserved genomic segment no. 7 [matrix protein (MP)-segment] of the viral genome is an attractive region for generic avian influenza detection by RT-qPCR (Spackman et al., 2002). The MP RT-qPCRs continue to be listed by the OIE for avian influenza detection in birds (WOAH, 2021). Both molecular and serology methods continued to be advanced and developed every year to increase the sensitivity and, in the case of avian influenza, broader application to all subtypes (Nagy et al., 2021). New techniques are designed to increase repeatability, reproducibility, and robustness of molecular diagnostics. This is crucial for influenzas in particular that are characterized by extreme genetic variability, circulating among different hosts. Connecting networks of pathogen surveillance teams will help ensure that the most sensitive and best diagnostic tests are being used and sharing information of new technological and analysis developments.

### 7.1 Reagents and laboratory supply chain

Not surprisingly, and especially since the COVID-19 pandemic, there have been issues with the supply chain for laboratory consumables and reagents in most, if not all, countries. This is especially true in countries that are dealing with other issues such as wars, environmental and natural disasters, or political unrest (Hilborne et al., 2022). Hilborne et al. (2022) point out that shortages of specimen tubes, PPE, and other common laboratory consumables threaten access to all aspects of diagnostic testing. Through dealing with this laboratory and health care crisis, laboratory medicine stewardship guidelines such as Choosing Wisely (Choosing, 2023) have been developed. These guidelines were designed around the patient-centric and fiscally prudent principle of reducing testing that adds no value to patient care, and that may even be associated with increased risks (Dickerson et al., 2017). It is important to note that although a call for increased biosurveillance in wild birds would inherently increase the use of laboratory supplies, reagents, and PPE, it is prudent to refer to laboratory stewardship guidelines to make sure that supplies are not wasted. After following stewardship guidelines to reduce waste, it is crucial to build shipment delays and sometime complete inability to obtain supplies in some regions into research project timelines. In some cases, collaborative networks can help with supply shortages or work to develop troubleshooting for swapping reagents or chemicals that have been tested to be valid.

### 7.2 Next generation sequencing and bioinformatics

Pathogen-specific PCR testing is fast and efficient for detecting and ruling out specific pathogens. However, identification of novel pathogens or pathogens without PCR primers requires next generation sequencing (NGS). Used in conjunction with conventional tests, NGS can provide additional information for outbreak response including pathogen emergence, evolution, and transmission (Lam et al., 2016; Lam and Pybus, 2018). NGS requires no prior knowledge of the pathogens being tested for and can be used to detect multiple pathogens at once from a variety of sample types (Himsworth et al., 2020). NGS can be used for whole genome sequencing of long-reads for organisms such as with the Minion or PacBio sequencers, or for metagenomic analysis of total microbes in sample. As seen during the COVID-19 pandemic, the use of sequencing and bioinformatics tools to track variants of SARS-CoV-2 is crucial for putting mitigation measures in place and determining sources and timing of transmission events. Likewise, for avian pathogens, whole genome sequencing can help identify and explain multiple subtypes of avian influenza or strains of bacterial pathogens along migratory pathways (Pearce et al., 2009). Such information is required to understand routes of transmission from wild birds to domestic animals, and potentially, to humans. Genomic data can also help to identify species to target for future surveillance efforts (Pearce et al., 2009).

Bioinformatics tools and pipelines are essential to quickly analyze genomic data for surveillance purposes. The process of going from sample to sequence includes DNA extraction, quality control, library preparation, and sequencing, which can take days to complete. On top of that, bioinformatics takes additional time and computational resources. The ability to take raw sequencing reads (.fastq files) and turn them into useable data with already developed tools can help provide useable information quickly. Many bioinformatics tools are useful for specific questions and applications, and using the appropriate tool required for the desired output will save time and resources. For example, identifying potential pathogens in a metagenomic sample requires different tools than if the goal was to characterize a pathogen using whole genome sequencing to determine to which strain(s) the pathogen is most closely related. Having pre-defined and streamlined tool pipelines will give the most appropriate results in the quickest time possible (de Vries et al., 2022).

One consideration when using sequencing in a surveillance approach is server storage space. A large volume of storage is needed to store large amounts of NGS data. Making sure there is enough space to store all sequencing data safely and securely can also provide faster and more informative results in the future as more samples are sequenced. For example, a database consisting of only pathogen genomes of interest can help decrease time needed for pathogen detection.

### 7.3 Maximizing viral diversity and yield

Currently, a diversity of HPAIs is circulating the globe along with a multitude of other less pathogenic subtypes in wild and domestic birds and mammals. As pointed out by Machalaba et al. (2015), a shift in screening practices is required to move beyond emphasis on HPAI viruses. NGS should now be a part of surveillance programs to provide molecular information on the different HA subtypes in influenza viruses. As in the recent reassortment of the HPAI H5N1, NGS reporting was a key factor in both understanding and raising concerns with the 2022–2023 H5N1 outbreak landscape and transmission cycle (Xie et al., 2023). In this case, a novel reassortment of (HPAI) (H5N1) clade 2.3.4.4b.2 was identified in dead migratory birds in China in November 2021 (Xie et al., 2023; Yang et al., 2023). Yang et al. (2023) hypothesize that the viruses probably evolved among wild birds through different flyways connecting Europe and Asia, which was confirmed then by an in-depth analysis by Xie et al. (2023). The western movement of clade 2.3.4.4b was quickly followed by additional reassortments with influenza viruses circulating in wild birds in North America (Kandeil et al., 2023).

When it comes to sampling smaller birds, environmental sampling, or sampling birds with lower viral loads, it is critical to maximize the viral RNA yield for molecular diagnostics and sequencing. First, extensive mechanical disruption for homogenization swabs in RNA Later and centrifugation will increase RNA yield. It is also important to use the correct amount of starting material per the specific protocol, to not overload, and to perform all protocol steps at room temperature. It is also important to not miss the dry-spin step prior to elution and to place the eluant onto the center of the membrane. As in common practice in molecular and sequencing laboratories, there is extraction of triplicate samples and elution in the same column.

## 8 Wild bird mortality events

Emergency preparedness plans for emerging and re-emerging deadly zoonotic diseases must be in place and continuously developed to allow countries to respond quickly to disease detection and be in a proactive situation instead of a reactive one. The occurrence of sudden bird deaths in a specific region in a relatively short time, especially with visible severe lesions on birds, should lead to two main questions: (1) Will this influence human health, and (2) what is risk for transmission to domestic poultry populations in the area?

People’s health and safety in responding to a mortality event are considered the priority, with following more stringent biosafety protocols for sampling at a wild bird mortality event. The first step would entail reporting to government officials, if applicable, and to remove animal carcasses in proper way, quarantine the affected farms in the area. Meanwhile, the importance of securing food chain sustainability in poultry or other potentially susceptible animals should considered. An impact analysis should consider not only the birds, but also other animals in the surrounding environment (soil, water, plants, and air) that might be affected by the disease, and in turn, any adverse effects on the availability of food supply for people, directly or indirectly.

The emergency preparedness and response plan for a newly emerged deadly zoonotic disease in birds must also include identifying the first responder authorities, organizing the reporting process (who will report to who and what will be reported), improving testing capabilities, gathering, and interpreting test information, and finally, communicating and publishing results. World Organization for Animal Health (WOAH) maintains a list of notifiable diseases that is updated annually for the WOAH member countries.

## 9 Regional and flyway surveillance networks

With the expansion of different HPAIs, establishing global research and coordination networks for strengthening capabilities throughout the world is needed to close the gap of zoonotic disease surveillance testing, to provide real-time information to support authorities, and to protect human and animal health. One example is the creation of threat reduction networks to build and strengthen a sustainable international community of biosecurity, disease ecologists, and biosurveillance experts to address shared biological threat reduction challenges (Ambrosiano et al., 2020). As called out by Machalaba et al. (2015), establishing collaborative networks across countries would “be cost-effective, reduce the need for additional laboratory capacity in regions of interest, and complement other surveillance programs.” Machalaba et al. (2015) also point out that a better understanding of viral diversity can be gained through earlier detection and surveillance of pathogen genes of both low-and highly pathogenic influenza. Coordinated research networks can create connected intelligence among researchers and institutions around the world and are central to the concept of cooperative threat reduction of zoonotic risks.

One of the challenges discussed by Lawson et al. (2021) is the difficultly in recruiting, training, and maintaining expertise in staff. Lawson et al. (2021) recommend communicating to governmental and university funders that it takes time to build the knowledge for wildlife health in a country. This includes communicating common interested and the benefits of working collaboratively across different sectors to build sustainable relationships and programs.

Government agencies and organizations are now actively seeking to foster and strengthen science networks, especially in global health. The return of investment for network-building activities is seen as an efficient measure for increasing research capacity and sustainability of connections (Nelson et al., 2018). While global health programs are establishing strong international collaborative research networks (Fair et al., 2016) through building capacity for surveillance research in partner countries through training (Johnson et al., 2015; Standley et al., 2015), there still needs to be more coordination between international agencies and established networks. For example, an international consortium for zoonotic infectious diseases in wild birds could help establish standards for metadata collected, field and laboratory protocols, and bioinformatic methods. International networks also offer support for increased biosafety and biosecurity guidance and resources in countries that support the International Health Regulations (Standley et al., 2015).

### 9.1 Avian zoonotic diseases and Black Sea Mediterranean flyway

**Figure 3:**
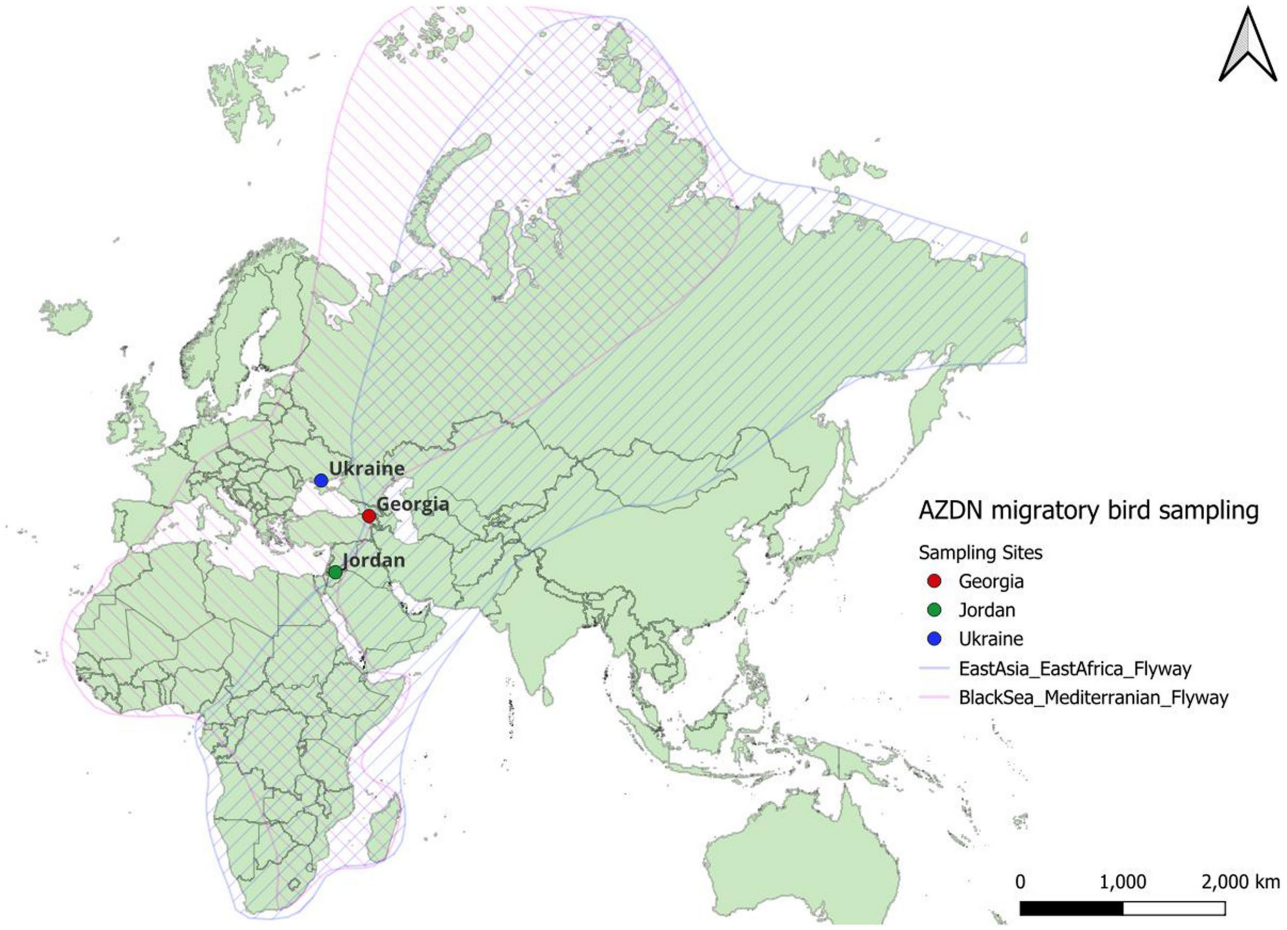
The East Asia Africa and Black Sea Mediterranean Flyways with the Avian Zoonotic Disease Network countries located.

The Black Sea-Mediterranean Flyway (MBSF) is important for migrating birds due to its strategic location and diverse ecosystems, serving as a crucial pathway for hundreds of wild bird species (Birdlife, 2023). The MBSF is one of three Palearctic-African flyways connecting wild bird summer grounds in Europe with African wintering grounds (Figure 3). This flyway is one of the world’s largest bird migration systems and has been found to be important for the movement of zoonotic pathogens by wild birds (Najdenski et al., 2018). It is thought that over 2.5 billion birds move through the MBSF every migration. The varied landscapes along this route, including wetlands, coastal areas, and diverse terrestrial habitats, offer essential stopover sites for migratory birds. These stopover areas provide crucial landscapes during migration for birds to rest and refuel. Moreover, the Black Sea-Mediterranean flyway plays a vital role in the conservation of numerous species, as it supports a high concentration of globally threatened and endangered birds. Efforts to preserve and manage key sites along this flyway are imperative for maintaining the health of bird populations, promoting biodiversity, and contributing to the overall functioning of ecosystems within this ecologically significant corridor. Providing landscapes that provide health in wildlife and are protected areas away from humans and agricultural animals can limit land use-induced spillover which is the process by which land use change drives the transmission of pathogens from wildlife to humans (Plowright et al., 2021).

While there are numerous examples of coordinated research networks with a focus on infectious diseases, here we highlight the cooperative threat reduction network, called the Avian Zoonotic Infectious Disease Network (AZDN) with a focus on the MBSF. The small, but connected AZDN supports the countries of Jordan, Georgia, and the Ukraine to both strengthen and develop monitoring efforts and within the MBSF. This threat reduction network brings together experienced avian disease researchers with lesser-experienced biologists to provide instruction on how to capture, handle, and sample wild birds, and how to go from sample to sequence for both common and potentially rare avian viruses, including avian influenza. Through this coordinated network, every aspect of the requirements described in this paper had to be completed—most prior to sampling. The challenges for coordinating this network included a major war, dealing with post-pandemic supply chain issues, and geopolitical unrest. Nonetheless, the AZDN was able to work through these major hurdles to establish wild bird monitoring along the MBSF.

### 9.2 Three country perspectives coordinating an avian disease surveillance program

#### 9.2.1 Ukraine

Selecting the most suitable location for active monitoring of zoonotic pathogens in wild birds is very important. Today, Ukraine is one of the important locations for active monitoring of wild bird influenza (Waldenström et al., 2022). Ukraine has a unique geographical location in Europe and is a bridge between Europe and Asia. Several transcontinental migration routes of wild birds of many species and ecological groups intersect there. Additionally, Ukraine has favorable natural conditions for year-round stay of wild birds of different ecological groups, as well as suitable conditions for migratory birds (for nesting, resting during migration and wintering). Almost the entire territory of Ukraine is favorable for wild birds, but the highest concentration of wild birds (especially waterfowl and wading birds), which are natural carriers of the influenza virus and other zoonotic diseases, is observed in the south of Ukraine (Kherson, Zaporizhzhia, Odesa, Mykolaiv regions, and the Autonomous Republic of Crimea).

Given the long-term ornithological observations (migration density, nesting, and wintering) and the many years of experience of the Ukraine National Scientific Center’s Institute of Experimental and Clinical Veterinary Medicine (NSC IECVM) from 2001 to 2023 for the monitoring of influenza and Avian orthoavulavirus in wild birds in Ukraine, there ample data on wild populations during the breeding season and migration (Muzyka et al., 2014, 2016, 2019). Furthermore, there is a large network of regional natural parks, national parks, nature reserves, and biosphere reserves in Ukraine engaged in active study and protection of wild birds that can be involved in the monitoring of pathogen circulation among birds. Access to these areas is supportive for scientific research. Also in Ukraine, there are no problems with access for bird research on private agricultural land and other private territories. Usually, access to these areas is allowed with the approval of the owners. The study of bird movements and migration is important for the research of avian disease ecology. Ukraine has a long tradition and history of bird ringing, operated by the National Bird Ringing Center, and has a large ringing database.

Approaches to the transportation of biological samples following biosafety and biosecurity requirements, and in compliance with cold chain have been developed and are actively implemented in Ukraine. Logistics in Ukraine is also well developed, with a wide network of state veterinary laboratories that can be involved in active monitoring as logistics centers for temporary storage of samples, if necessary. The leading role in wild bird monitoring belongs to the NSC IECVM, which engages other research, diagnostic and educational institutions in its investigations.

As of 2023, there are now grave concerns for the safety of research in Ukraine. Before the onset of the war in Ukraine in 2014, the entire territory of Ukraine was completely secure for wild bird research. After Russia’s illegal annexation of the Autonomous Republic of Crimea and parts of Donetsk and Luhansk oblasts in 2014, research in the Autonomous Republic of Crimea and the eastern coast of the Sea of Azov has become temporarily impossible. Following the large-scale Russian invasion of Ukraine in February 2022, some of the main locations for active wildlife monitoring in the south are under temporary occupation (parts of Kherson and Zaporizhzhia regions, the Autonomous Republic of Crimea), where activities are also currently impossible.

At the same time, the main locations of wild bird concentration in the southwestern region of Ukraine on the Black Sea coast are accessible for scientific research with some restrictions (necessity to receive prior approval of research areas from local authorities and the military). In general, the logistics of research in this region is also well established. Ukraine continues to consider the monitoring of avian populations and zoonotic pathogen surveillance of great importance for the country and region and is dedicated to doing this work safely and securely.

#### 9.2.2 Georgia

Georgia is situated on the eastern shore of the Black Sea. Three waterfowl migratory flyways overlap in the Caucasus Region. Georgia is in the very center of the Black Sea/Mediterranean flyway that covers most of central and Eastern Europe. Georgia is also situated within the western border of the flyway West Asian/East African flyway that along the Eastern borders of the European Union. The western border of Central Asian flyway also encompasses the easternmost parts of Caucasus of Georgia.

The most important wetland regions in the country are the Javakheti Uplands and the Black Sea coast (Lewis et al., 2013; Venkatesh et al., 2018; Waldenström et al., 2022). The Javakheti Upland harbors six out of the eight largest natural lakes of Georgia. The lakes are shallow, rich with microplankton, and aquatic water vegetation. The lakes are situated at high altitudes (1,800–2,200 m above sea level) and are important migratory stopover sites for waterbirds passing through the Caucasus during both spring and autumn migration. Same lakes are also most important waterfowl breeding sites. Javakheti upland lakes are used by tens of thousands of waterfowl from Siberia for the migration stopover, but lakes are usually frozen from December to April, and all waterbirds leave the upland for wintering to the west and south.

For this project, Madatapa Lake was selected as a main field sampling location. Madatapa Lake is situated in the Javakheti Uplands at an altitude of 2,100 m above mean sea level. The lake regularly harbors several thousands of migratory waterbirds, especially mallard (*Anas platyrhynchos*), Northern pintail (*Anas acuta*), Eurasian teal (*Anas crecca*), and garganey (*Spatula querquedula*).

To capture the full picture of disease dynamics in migratory bird populations, we have selected most important over-wintering lake of eastern black sea, as our second sampling site. Paliastomi Lake is situated on the central part of eastern Black Sea coast and this coastal lake is a part of a system of the larger Colchic wetlands. Paliastomi Lake, together with the surrounding wetlands around the Black Sea are internationally important waterbird overwintering sites. The Eastern Black Sea coast is also an important migratory corridor for other avian species as well. Hundreds of thousands of passerines and over a million raptors migrate through the corridor.

The Center for Wildlife Disease Ecology (CWDE) at Ilia State University started active surveillance of AIV in wild birds in 2010. Over the past decade, the surveillance system has been continuously refined based on annual results and focused on understanding the ecology and evolution of AIV within wild waterbirds. As a result of accumulated experience, in 2015, the CWDE constructed and operated a duck funnel trap based on a Swedish design from Ottenby (Bub, 1991). The trap captures predominantly wild waterfowl and is located on the Shore of Madatapa Lake.

The CWDE has been doing active surveillance of AIV at the Paliastomi Lake from 2010. Since 2014, we have used clap nets to sample several species of gulls and shorebirds (Bub, 1991; Tulp and Schekkerman, 2001). During our current study, and in the past, environmental (fecal) sampling of gull flocks has been used. When the operation a clap net is not possible due to challenging circumstances, using the clap trap method is an effective approach. Previous work carried out in Georgia, where longitudinal AIV surveillance has been undertaken was critical for understanding ecology, epizootic dynamics, viral diversity, evolution, and movement of AIV (Lewis et al., 2013; Venkatesh et al., 2018; Waldenström et al., 2022). Capturing regularities in such complex systems as host-disease interaction, is necessary for increasing the predictive power of disease spread models.

#### 9.2.3 Jordan

Every spring and autumn, more than 500 million birds fly through the Middle East and North Africa, to breed or escape the winter months. Jordan lies on one of the most vital bird migration paths in the world, through which birds from Europe pass during the spring and autumn migratory seasons. Due to dry desert conditions, oases and irrigated olive and fruit plantations in or at the edge of desert areas can be rather attractive for migratory passerines. Previous reconnaissance visits by Jordanian ornithologists or previous experience with individual sites in both migration seasons recommended the density of passage migrants stopping over may differ significantly between spring and fall migration seasons. As of 2019, over 440 bird species have been identified in Jordan, more than 300 of which are migratory and pass through the Kingdom on their way from Europe to Africa and vice versa.

In Jordan, as in all locations for capturing wild birds, its important sites with mist nets should be protected from large animals, domestic and feral cats. It is beneficial if the study sites are fenced areas like a reserve or private land. The experience with shorebirds in Jordan and other arid areas showed that best sites are those having a combination of properties. Suitable sites for shorebirds in Jordan are heterogeneous wetland habitat with (partly) flooded mudflats neighboring ponds with shrubs and reeds, where there are local concentrations of shorebirds during migration. Along and near these edges, i.e., between the open mudflat and other shallow ponds, which are partly surrounded by shrubs, shorebirds seem to be moving a lot and less aware of the mist nets and are thus easier to catch than in completely open and homogeneous mudflat. In this respect, the Azraq Wetland Reserve and Aqaba Observatory are the only two potential sites for capturing and sampling migratory birds. Both sites are operated by the Royal Society for the Conservation of Nature (RSCN). Although the Aqaba Observatory in the south might be a good site for capturing migratory birds (mainly gulls and ducks), and/or at least collecting environmental samples from the shore, this site is located near the border and other military bases, and thus challenging for access and approvals.

To access the study sites in Jordan, the following documents are needed: (1) a permission letter from the Ministry of Environment to collect migratory bird samples in Jordan, and (2) a from RSCN that we can access the RSCN sites “Reserves” for collecting migratory bird samples. We were not successful in catching shore birds using mist nets in open and homogenous habitat, even if bird density was relatively high. Mudflats must contain big areas of shallow water; moist mud alone seems to be attractive only for a few species like Kentish plovers (*Charadrius alexandrines*). It is more likely to trap shorebirds at sites and at times with high density and diversity of shorebirds at dawn until sunrise and around sunset.

The waterfowl, shorebird, and passerine capturing approaches for pathogen monitoring in migratory birds was introduced and built-up for the first time ever in Jordan. Currently, the team is using swim-in and confusion duck traps inside two oases at Azraq Wetland Reserve, as well as mistnets for shorebirds and passerines. The team decided to use these traps for sentinel ducks (domestic ducks used to be exposed to other wild ducks). A special IACUC was developed and approved for using, handling, and sampling sentinel ducks in Jordan. Sampling is limited to spring and fall migration. The confusion trap was set up on the ground near the water, where the ducks usually rest and sleep after sunset until sunrise, and where the bait is always available. We found this approach more attractive and promising for capturing wild ducks to fulfill the project’s requirements. Environmental samples can be collected both inside and around the trap during the hold-on period, as well. Likewise, environmental samples have been collected from bird colonies in Jordan.

##### 9.2.3.1 Building capacity for sustainable monitoring of migratory wild birds

In building the capability for capturing birds in Jordan, that did not have long-term monitoring in place, perseverance for learning to with the local habitat situation was imperative. With multiple unsuccessful trials at a location, the team must be ready to try out many things until they succeed and improve their trapping rates for migratory birds. As conditions vary among sites and countries, there are always certain things to consider and there cannot be one unified recipe for all. As in any country’s sampling of wild birds there are many different roles for team members carrying out trapping, bird identification and ringing and sampling. It is important to work with local ornithologists who can identify and ring the birds. Experienced ringers who are present in the field should be given freedom in training and supervising personnel with less experience in taking birds out of the nets and handling birds in general. Likewise, ornithologists may learn more about sampling and disease ecology in this exchange of knowledge and experience. However, teaching younger trainees in bird identification and the aging and sexing of individuals may take years and require hundreds of birds and trapping/ringing hours.

With new countries just developing wild bird monitoring and pathogen surveillance, the knowledge exchange is crucial for the building of the multidisciplinary team that may come from different backgrounds in biology. This openness in knowledge exchange will help build trust within the team that may be working long hours in adverse field conditions. It is helpful that all team members can gain experience for each of the different roles of setting mist nets or traps, collecting and handling birds, sampling the birds, data collection, and storing the samples and supplies. While learning new techniques and research experience in an entirely new field can be challenging, this how a team can grow together to build a strong avian disease monitoring program.

## 10 Lessons learned and future directions

There are lessons to be learned from the experience of strengthening and coordinating wild bird disease surveillance in three countries along a migratory flyway. Building an international research collaboration network is challenges in the best circumstances, but even more difficult with the backdrop of a pandemic, geopolitical politics and war, and the mis and disinformation age for science. Having a global network of networks that researchers around the world could tap into for resources, information, data, and general support for the biosurveillance in wild birds would ensure that the best methods are being used and limited resources are put to the best use. Table 1 identifies the key knowledge gaps and immediate needs for strengthening surveillance efforts for wild birds safely and securely around the world.

**Table 1:** Key recommendations to strengthen surveillance of wild birds and collaboration.

Knowledge gap or need	Recommendation
Capability to safely capture and handle wild birds for sampling	More wild bird capture and handling trainings for wildlife biologists and connection of ornithologists with infectious diseases specialists.
Biosafety in the field	Trainings for ornithologists for capturing of birds that have increased likelihood of infections or near outbreak areas.
Trust in multidisciplinary teams	Focus on trust building between laboratory specialist and ornithologists is imperative.
Hypothesis-testing to understand the ecology of infectious diseases in wild birds	Education on the gaps of understanding on the ecology of host/pathogen/vector systems and additional samples or data systematically collected from birds to answer key questions.
Sample and data sharing	Connection of laboratory and ornithologists for additional analysis of DNA/RNA or other analysis. Sharing of data and including more metadata in publications. Protocols for safe shipment of samples or extracted DNA/RNA.
Environmental and climate drivers of zoonotic diseases in wild bird populations	Connection between ornithologists, disease specialists, and climate scientists to use surveillance data and analysis to forecast future outbreaks and pathogen range shifts.
Microbial evolution	Connection with surveillance programs to viral and bacterial bioinformatic and evolution specialists and availability to sequencing.
Wild bird conservation	Inclusion and outreach to conservation organizations and governments to understand the impacts of diseases on wild bird populations globally and local populations.
Impact and risks to poultry	Better connection between wild bird surveillance and the poultry industry and government authorities to access and know of disease risks.
Education and outreach	Connection to public communication experts to design educational information for public on wild birds and ecology of disease, especially to correct mis/disinformation.
Communication with all stakeholders	Increased transparency with all government authorities, organizations, or entities that can assist in surveillance or have a need to know of results. Trainings for all personnel involved in surveillance to ensure rigorous scientific method and data quality to trust the results.

A One Health approach requires collaboration between wildlife, veterinary and human health sectors. Collaboration is necessary not only on the national level, but internationally between different countries as well. Sharing of the information from different geographic locations in Eurasia, can be only achieved by constructing effective collaborations between teams working on the ground at different key locations. Below are additional lessons learned from all the three countries that include having both long-term monitoring experiences and are newly establishing a surveillance program.

1. Sufficient and specific ornithological expertise is crucial for the success of sampling wild birds. Even if there has been no previous sampling of wild birds, including ornithologists is vital to begin a sampling program. This will ensure that animals will be captured and handled with safest methods, identification of species, sex and age will be done correctly, and that relevant ecological information is collected on the field.
2. The avian capture team should undertake training in all aspects of the surveillance that include, biological sampling techniques, biosafety procedures, safe sample handling, storage, and transportation of sampling.
3. Good direct working relationship and communication between field and laboratory teams is of great importance for ensuring more precise and fast data generation. Providing opportunities for the field and laboratory personnel to interact and exchange ideas will help foster trust and communication between the different roles in a surveillance program.
4. Low personnel turnover rate in the team should be achieved to ensure effectiveness of work.
5. From the start of the project ensure discussion of both ornithology and virology experts for the selection of sites and the development of field methodologies.
6. All permits and ethical documentation should be considered and prepared from the very beginning of the project, so no unexpected delays are kept at a minimum.
7. Connecting to other ornithologists and surveillance teams can provide opportunities for asking questions outside of the immediate team and offer new insights for standards for zoonotic pathogen surveillance in wildlife.

As more researchers and agencies sample wild birds for zoonotic pathogens around the world, the better the understanding will be for ecology of these infectious diseases on a global scale. Likewise, as Machalaba et al. (2015) point out, shifting away from only screening for highly pathogenic avian influenzas to all influenzas (and other pathogens), may provide more robust subtype findings. Surveillance efforts and teams sampling wild birds exist around the world and may be connected via scientific reports or conferences. Laboratory networks and reporting government organizations may be connected via OIE reporting requirements. However, creating a network of connected scientists and researchers from academia, industry and government working at the forefront of wild bird pathogen surveillance, could provide an invaluable resource for all countries to safely sample wild birds and maybe at the same time, better understand avian populations use the landscapes around the world. We echo the words and call of Plowright et al. (2021) for “colleagues across the fields of environmental, wildlife, and human health to forge the collaborations urgently needed to advance our knowledge of how land use change drives zoonotic disease emergence.” Through a more connected network for wild bird surveillance, better coordination can take place between researchers and the reporting agencies and international organizations.

To optimize wild bird surveillance globally, we recommend greater coordination across networks or organizations or a creation of conference on wild bird surveillance. With limited resources for global One Health biosurveillance overall, increased efforts to share information, expertise, and data could assist in creating better surveillance, that is more sensitive and ensure the safe handling of birds and samples. Lastly, collecting additional data on bird health and condition, life history traits, environmental variables, and weather conditions can provide insights and increased understanding of the ecology of pathogens in migratory birds that will better inform biosurveillance and mitigation efforts.

## Author contributions

JF: Conceptualization, Data curation, Formal analysis, Funding acquisition, Investigation, Methodology, Project administration, Resources, Supervision, Validation, Visualization, Writing – original draft, Writing – review & editing. NA: Conceptualization, Funding acquisition, Investigation, Resources, Supervision, Writing – original draft, Writing – review & editing. MA: Conceptualization, Investigation, Methodology, Supervision, Writing – original draft, Writing – review & editing. AB: Conceptualization, Formal analysis, Funding acquisition, Investigation, Methodology, Project administration, Resources, Supervision, Writing – original draft, Writing – review & editing. SB: Conceptualization, Investigation, Methodology, Writing – review & editing. ID: Conceptualization, Investigation, Methodology, Visualization, Writing – review & editing. AE: Conceptualization, Funding acquisition, Investigation, Project administration, Resources, Writing – original draft, Writing – review & editing. LF: Conceptualization, Investigation, Project administration, Writing – review & editing. ZJ: Conceptualization, Formal Analysis, Investigation, Methodology, Project administration, Visualization, Writing – original draft. FK: Conceptualization, Data curation, Investigation, Methodology, Supervision, Validation, Writing – original draft. DM: Conceptualization, Data curation, Funding acquisition, Investigation, Methodology, Project administration, Resources, Supervision, Validation, Writing – original draft, Writing – review & editing. LN: Writing – review & editing. JT: Conceptualization, Data curation, Funding acquisition, Investigation, Project administration, Supervision, Writing – original draft, Writing – review & editing. LU: Conceptualization, Funding acquisition, Investigation, Methodology, Project administration, Resources, Supervision, Writing – original draft, Writing – review & editing. JO: Conceptualization, Data curation, Funding acquisition, Investigation, Methodology, Project administration, Resources, Supervision, Writing – original draft, Writing – review & editing.
